# Supplementary material for: Enhancement of antifungal activity and transdermal delivery of 5-flucytosine via tailored spanlastic nanovesicles: statistical optimization, in-vitro characterization, and in-vivo biodistribution study
Source: Front Pharmacol. 2023 Dec 4;14:1321517. doi: 10.3389/fphar.2023.1321517 (PMC10731591; doi:10.3389/fphar.2023.1321517)
Supplement: Supplementary file 1 [file Table1.DOCX]

**Enhancement of antifungal activity and Transdermal delivery of 5-flucytosine *via* Tailored Spanlastic Nanovesicles: Statistical Optimization, In-vitro characterization, and In-vivo Biodistribution Study**

Awaji Y. Safhi^1^, Nimbagal Raghavendra Naveen ^2^, Krishna Jayanth Rolla ^3^, [Penmetsa Durga Bhavani](about:blank)^4^, Mallesh Kurakula^5^, Khaled M. Hosny^6*^, Walaa A. Abualsunun^6^, Mohammed Alissa^7^, Abdullah Alsalhi^1^, Amerh Aiad Alahmadi^6^, Khalid Zoghebi^8^, Abdulrahman Sindam Halwaani^9^, Rasha Ibrahim K^10^

^1^ Department of Pharmaceutics, College of Pharmacy, Jazan University, Jazan 45142, Saudi Arabia; [asafhi@jazanu.edu.sa](about:blank) (A.Y.S); [aalsalhi@jazanu.edu.sa](about:blank) (A.A);

^2^ Department of Pharmaceutics, Sri Adichunchanagiri College of Pharmacy, Adichunchanagiri University, B.G Nagara, Karnataka 571448, India. [raghavendra.naveen@gmail.com](about:blank) (N.R.N)

^3^Department of Biotechnology, Sri Indu Institute of Engineering, Ibrahimpatnam, Hyderabad, Telangana, India; [jayanth.r07@gmail.com](about:blank) (J.)

^4^Department of Pharmaceutics, Vishnu Institute of Pharmaceutical Education and Research, Telangana, India; [durgabhavani.p@viper.ac.in](about:blank) (P.D.B)

^5^Thermo Fisher Scientific, Bend, OR 97701, USA. Email: [mallesh_kurakula@yahoo.com](about:blank) (M.K)

^6^Department of Pharmaceutics, Faculty of Pharmacy, King Abdulaziz University, Jeddah 21589, Saudi Arabia; [kmhomar@kau.edu.sa](about:blank) (K.H); [wabuassonon@kau.edu.sa](about:blank) (W.A.A); [mamalahmadi@kau.edu.sa](mailto:mamalahmadi@kau.edu.sa) (A.A.A)

^7^Department of Medical Laboratory Sciences, College of Applied Medical Sciences, Prince Sattam bin Abdulaziz University, Al-Kharj 11942, Saudi Arabia; [m.alissa@psau.edu.sa](about:blank) (M.A)

^8^Department of Pharmaceutical Chemistry, College of Pharmacy, Jazan University, Jazan 45142, Saudi Arabia; [Kzoghebi@jazanu.edu.sa](about:blank) (K.Z)

^9^Central lab for neurosciences, King Abdulaziz University, Jeddah 21589, Saudi Arabia. [Amalsindi2020@gmail.com](about:blank) (A.S.H)

^10^Department of Pharmaceutics and industrial pharmacy, Faculty of Pharmacy, Beni-Suef University, Beni-Suef, Egypt. [Rasha_khallaf@yahoo.com](about:blank) (R.K)

***Corresponding Author:**

Dr Khaled Hosny

Department of Pharmaceutics, Faculty of Pharmacy, King Abdulaziz University, Jeddah, Saudi Arabia

Mobile: 00966561682377

Email: elswaify2000@yahoo.com

**Enhancement of antifungal activity and Transdermal delivery of 5-flucytosine *via* Tailored Spanlastic Nanovesicles: Statistical Optimization, In-vitro characterization, and In-vivo Biodistribution Study**

**Abstract**

**Aim and Background**: This current study aimed to load 5-flucytosine (5-FCY) into spanlastic nanovesicles (SPLNs) to make the drug more efficient as an antifungal and also to load the 5-FCY into a hydrogel that would allow for enhanced transdermal permeation and improved patient compliance.

**Methods**: The preparation of 5-FCY-SPLNs was optimized by using a central composite design that considered Span 60 (X1) and the edge activator Tween 80 (X2) as process variables in achieving the desired particle size and entrapment efficiency. A formulation containing 295.79 mg of Span 60 and 120.00 mg of Tween 80 was found to meet the prerequisites of the desirability method. The optimized 5-FCY-SPLN formulation was further formulated into a spanlastics gel (SPG) so that the 5-FCY-SPLNs could be delivered topically and characterized in terms of various parameters.

**Results**: As required, the SPG had the desired elasticity, which can be credited to the physical characteristics of SPLNs. An ex-vivo permeation study showed that the greatest amount of 5-FCY penetrated per unit area (Q) (mg/cm^2^) over time and the average flux (J) (mg/cm^2^/h) was at the end of 24 hours. Drug release studies showed that the drug continued to be released until the end of 24 hours and that the pattern was correlated with an ex-vivo permeation and distribution study. The biodistribution study showed that the 99mTc-labeled SFG that permeated the skin had a steadier release pattern, a longer duration of circulation with pulsatile behavior in the blood, and higher levels in the bloodstream than the oral 99mTc-SPNLs. Therefore, a 5-FCY transdermal hydrogel could possibly be a long-acting formula for maintenance treatment that could be given in smaller doses and less often than the oral formula.

**Keywords:** 5-flucytosine, Nanospanlastics, Optimization, Gel, Health care, Biodistribution, Antifungal

**1. Introduction**

*Candida albicans* is an opportunistic fungal pathogen that has significant effects on human health; it accounts for 6.8% of hospital-acquired infections in the United States [1]. Despite the availability of several types of antifungal medications, the toll from the deaths it causes, the financial burden associated with its use, and the prolonged hospital stays resulting from invasive candidiasis remains unacceptably high [2]. Furthermore, there is growing concern about the emergence of resistance to the existing antifungal drugs [3]. Hence, the quest for novel targets for antifungal treatments remains paramount [4]. *C. albicans* poses many threats owing to its ability to release aspartyl proteases and lipases, form filamentous structures, and establish robust biofilms [5]. A more profound comprehension of the biology and regulatory mechanisms governing these processes and pathways holds the potential to unveil innovative avenues for antifungal therapies [6].

The transdermal route seems a good choice owing to its non-invasiveness and the avoidance of the intrinsic metabolic processing of a drug [7]. Other benefits of this route are that it allows for the slow sustained release of a drug and improves patient compliance [8]. Nanosystems for drug delivery and nanomedicines are scientific applications that are still quite new, but their use is rapidly expanding. Nanoscale tools solve problems and deliver therapeutic agents to the right places efficiently. Nanotechnology has made a big difference in improving the health of people with long-term illnesses by getting medicines to the right places at the correct times. Nanomedicine has many novel applications for delivering various drug moieties, including biological agents, immunotherapeutic agents, chemotherapy drugs, and many more [9].

Kakkar and Kaur transformed spanlastics, which are composed of flexible nanovesicles crafted from surfactants. These structures include a nonionic surfactant and an edge activator. The surfactant molecules are arranged as a dual-layered barrier enveloping the active agent. The presence of the edge activator reduces the inter-membrane pressure, leading to the breakdown of the vesicular system’s double membrane. As the membrane disintegrates,, the vesicular system becomes more pliable and capable of changing shape [10]. In recent years, there has been more interest in using spanlastics to improve drug transport along transtympanic, transungual, ocular, and topical routes. These flexible nanospanlastic vesicles have been a key part of improving external drug delivery, allowing for better drug penetration and controlled release of lipophilic and hydrophilic drugs over long periods. This, in turn, has made it easier for patients to follow their treatment plans, improved the effectiveness of treatments, and cut down on side effects. Ultraspanlastics can spread through the intracellular spaces of the stratum corneum and other layers of skin because they are so flexible. This makes it easier for drugs to get into the target dermal tissues quickly and increases the transdermal drug saturation. Spanlastics are very stable, especially compared with other liposomal forms. They are better able to carry medicine because they are very flexible and easy to shape. Also, unlike different formulations with cationic surfactants, they do not make patients feel uncomfortable.

Several authors found that to get the required product profile, the formulation factors had to be optimized, with a focus on meeting the desired quality standards [11–14]. The quality by design (QbD) method is the best way to ensure that all formulation factors significantly affect the process. QbD differs from the standard “one factor at a time” method because it makes the optimization process more efficient and cost-effective. Sreeharsha et al. (2020) stated that the QbD method may need more than one meeting to determine how different factors relate [15,16]. However, it gives a complete picture of the process and helps get the desired results while minimizing costs, time, and resources. This study shows how the QbD method created links between formulations and process variables by following International Conference on Harmonization (ICH) recommendations and using statistical software tools. This work aimed to find a way to deliver 5-flucytosine (5-FCY) through the skin by using highly flexible nanovesicles made from detergents mixed into a gel. This method was meant to help the body absorb drugs better in the treatment of *C. albicans* infections.

**2. Materials and Methods**

**2.1. Materials**

5-FCY was purchased from Sigma Aldrich (St. Louis, Missouri, USA). Yarrow Chem Products (Mumbai, India) gave us sorbitan monostearate (Span 60), polyoxyethylene sorbitan monooleate (Tween 80), polyvinyl alcohol (PVA), methanol, and pure alcohol (97%). Spectrum Laboratories, Inc. in Rancho Dominguez, California, USA, provided a semipermeable membrane tube with a molecular weight cutoff (MWCO) of 12,000 to 14,000.

**2.2. Fourier-transform infrared (FT-IR) spectroscopy**

FT-IR tests were performed to determine if 5-FCY was chemically compatible with the other ingredients in the mixture. Solid samples were mixed with potassium bromide (KBr) and compressed into compact discs. As such, the liquid samples were looked at. An FT-IR spectrophotometer (IRSpirit, Shimadzu, Japan) was used to scan the solid and liquid samples in the transmission mode, covering 4000 to 400 cm^-1^ [17].

**2.3. Preparation of 5-FCY‒loaded spanlastics (5-FCY-SPLs)**

As previously described by Sallam et al. (2020), the ethanol injection method was employed to prepare spanlastic dispersions loaded with 5-FCY [18]. In brief, 5-FCY and Span were dissolved in ethanol maintained at 50°C. The solution was then slowly injected into a preheated aqueous solution (60°C) containing an edge activator (Tween 80). The resulting mixture of water and alcohol was placed on a magnetic mixer (IKA, Cole-Parmer, Wertheim, Germany) and vigorously stirred until all the alcohol had evaporated. Dialysis was performed to remove any excess drug, following the procedure outlined by Machado et al. in 2018. In summary, 3 mL of 5-FCY‒loaded spanlastic dispersions was placed into a dialysis bag (Sigma Aldrich; the bag had a molecular weight cutoff of 2 kDa), sealed at both ends, and immersed in 200 mL of water while agitated. Labraso was added as a permeation enhancer. Samples were collected from the receiving medium at regular intervals and analyzed for their drug content using UV spectroscopy at 243 nm, and this continued until no further drug was detected. The formulated products were then stored in amber-colored glass containers so they could be used for further research.

The preparation of the 5-FCY-SPLs was optimized using a statistical approach. The independent variables considered included the concentrations of Span 60 (X1) and Tween 80 (X2). The low, medium, and high factorial levels of each of these variables were denoted by the codes ‒1, 0, and +1, respectively. The dependent variables were the particle size (PS; variable Y1) and entrapment efficiency (EE; variable Y2) (Table 1).

**Table 1.** Central composite design of optimization for 5-FCY-SPLs

|  | Level used, actual and coded | | |
| --- | --- | --- | --- |
| Independent variables |  |  |  |
|  | **Low (-1)** | **Medium (0)** | **High (+1)** |
| X1 = Span 60 (mg)  X2 = Tween 80 (mg) | 100  80 | 200  100 | 300  120 |
| Dependent variables |  |  | **Goal** |
| PS (Y1)  EE (Y2) |  |  | Minimize  Maximize |

**2.4. Determination of drug content and EE of 5-FCY-SPLs**

Methanol was selected as the appropriate solvent for breaking down the prepared SPLs to determine the total drug content, as described in reference (31). To evaluate the total drug content, which encompassed the content of both the unentrapped drug and entrapped drug, 0.2 mL of a nanospanlastic dispersion was dissolved in 25 mL of methanol. Subsequently, the drug content was measured using a validated high-performance liquid chromatography (HPLC) method at 260 nm [19]. The entrapment efficiency percentage (EE%) of the 5-FCY‒loaded SNVs was determined using an indirect method involving ultracentrifugation to separate the unentrapped drug. Specifically, 1-mL samples of 5-FCY-loaded SPLs were centrifuged at 15,000 rpm for 1 hour at 4°C, utilizing a cooling centrifuge from Hermle Labortechnik GmbH (Wehingen, Germany). The supernatant was separated and filtered through a nylon membrane filter (Nylon Acrodisc, 0.20 μm, Gelman Sciences Inc., Ann Arbor, Michigan, USA). HPLC determined the concentration of free 5-FCY at 260 nm. The enclosure was found to be

*EE(%) = (A1 ‒ A2)* X$\frac{100}{A1}$

where A1 is the initial amount of drug and A2 is the amount of free drug in the supernatant.

**2.5. Determination of PS, PDI, and ZP**

The study also assessed the polydispersity index (PDI) and the zeta potential (ZP). To determine the average PS, PDI, and ZP, we employed a Zetasizer (Malvern Instruments Ltd., Malvern, Worcestershire, UK) set at a temperature of 25°C. The measurement was made after diluting the samples with double-distilled water to achieve the optimal scattering intensity. The PDI was used to indicate the similarity in PSs within the samples. We followed the method outlined by Kurakula et al. [20] to measure the zeta potential; this involved observing how the vesicles moved in response to an electric field in double-distilled water. We performed three replicates for each sample, and each replicate was measured twice to ensure accuracy.

**2.6. Preparation of** **5-FCY-SPLs containing a hydrogel (****SFG)**

The SFG was made by slowly adding 250 mg of hydroxypropyl methylcellulose (HPMC) K4M, which has a viscosity of 4000 cp, to 10 ml of magnetically stirred SF dispersion. The final HPMC concentration was 2.5% w/v [21]. Later, the mixture was put in the refrigerator and kept there until no air bubbles or noticeable clumps were present.

**2.7. Elasticity determination**

The extrusion method was used to test the elasticity of the optimized composition (SFG). Using tools from HAUG Sauer Kompressoren AG (St. Gallen, Switzerland) and Buchi Labortechnik AG (Flawil, Switzerland), 1 g of the prepared colloidal dispersion was forced through a 0.2-mm nylon filter for 3 minutes at a constant pressure of 2.5 bar. The following method was used to determine the deformability index (DI):

***DI =*** *j (* $\frac{rv}{rp}$*)*

where j is the weight of the sample (g) extruded, rv is the size of the spanlastics after extrusion (nm), and rp is the pore size of the nylon filter (nm) [21].

**2.8. Ex-vivo permeation study**

***2.8.1. Determination of equilibrium solubility of 5-FCY in 0.03% v/v lactic acid solution***

A specific amount of permeation medium was needed to find out how well 5-FCY dissolved in balance and ensure that the sink conditions stayed the same. In this case, about 10 ml of 0.03% v/v lactic acid solution with too much 5-FCY was put into glass bottles with tight-fitting caps. Then, for 48 hours, these bottles were moved around in a shaking water bath (GFL Gesellschaft für Labortechnik, GmbH, Burgwedel, Germany) set to 50 strokes per minute and 37°C. Then a 0.45-µm membrane filter was used to separate the solution carefully. After the right amount of diluting, spectrophotometric analysis was done of the resulting filtrate at the set peak wavelength of 276 nm. This experiment was done thrice to ensure the results were correct and consistent [22].

For the ex-vivo study, the skin of a goat was preserved in Tyrode solution (sodium chloride 8 g/l; potassium chloride 0.2 g/l; calcium chloride 2 H_2_O 0.134 g/l; sodium bicarbonate 1.0 g/l; sodium dihydrogen phosphate 0.05 g/l; and glucose H_2_O 1.0 g/l) until needed for experimental use. Before the experiment, the skin was left at room temperature for 2 hours with phosphate buffer water. The skin was then put in a plastic dialysis tube with the stratum corneum facing the donor compartment and the dermis facing the receiver compartment (n = 3 per formulation). The area that could be permeated was approximately 1.5 cm^2^. A volume of the formulation equal to 5 mg of 5-FCY was added to the donor compartment. The permeation medium was 30 ml of lactic acid solution at 37 ±0.5°C and 100 rpm. A pilot study showed no sign of drugs for up to 2 hours. Therefore, 0.5-mL samples were taken at 2, 4, 8, 12, 24, 36, and 48 hours, and they were immediately replaced with fresh lactic acid solution to keep the volume and sink conditions the same. The drug concentration was then measured.

**2.9. In-vitro release of 5-FCY from SFG and kinetic analysis of release data**

Wet tubes made of Spectra/Por semipermeable material and having a length of 5 cm (Spectrum Laboratories Inc., Rancho Dominguez, California, USA) were filled with the optimized formula. The tube samples were then put in 50 ml of a releasing medium (0.03% v/v lactic acid solution) in amber-colored glass bottles, which were sealed and shaken 50 times per minute for 24 hours at 37°C. At different times, aliquots of 1 ml were taken out and immediately replaced with fresh medium. Samples were collected three times and calculations were made of the average and standard deviation. Several models, such as the zero order model, first order model, and diffusion-controlled Higuchi model, were used to find the right kinetic model for the in-vitro release data. The coefficient of determination (R2) was calculated, and the model with the best R2 number was chosen as the release model.

**2.10. Rheological studies**

The gel was tested with a Brookfield spinning cone and plate viscometer with spindle CPE-41 (Brookfield Engineering, Middleboro, Massachusetts, USA). The gel had been made at a temperature of 25 ± 1°C. About 0.5 g of the test mixture was placed on the plate. The spinning speed varied from 0.5 to 100 rpm, with a 10-second break between each change. The data for viscosity and shear rate were thought to be valid only when the torque was between 10% and 100%. A graph was made to show how the viscosity and shear rate changed with the amount of shear force. The power law model was used to examine how the SFG behaved from a rheological point of view.

τ = ${\boldsymbol{K}\gamma}^{n}$

where**τ** represents the shear stress, **γ** represents the shear rate, ***K*** is the consistency index, measured in units of s, and ***n*** is the flow index, which is dimensionless.

Gad et al. (2008) stated that the value of n for a shear-thinning fluid is usually between 0 and 1. For a Newtonian system, n equals 1, and for a dilatant system, n is greater than 1 [23]. Different equations, such as the Bingham, Casson, and Carreau equations, are used to explain systems that are not Newtonian. By comparing the R2 numbers, we could determine the kind of system.

**2.11. In-vivo biodistribution study**

For the in-vivo biodistribution study, the animal model agreed with the plan for the trial. The animal study protocol was approved by the Animal Ethics Committee of Cairo Agriculture for Experimental Animals, Cairo, Egypt, Approval No. 05-07-23. Each plastic box contained five Swiss albino mice. The weight of each mouse was between 22 and 27 g. They were kept in a controlled environment at a temperature of 25.0 ± 0.5°C, a humidity of between 45% and 55%, and a 12-hour light and dark schedule. The mice could always get food and water [24].

***2.11.1. Preparation of radiolabelled SF and SFG***

SFG was diluted by adding 200 µL of buffer solution at pH 1.2 and enough distilled water to make a 1-ml sample. . Technetium-99m (99mTc) was used to make a reduced SFG (dil-SFG) that was radioactive. To perform radiolabelling of the substance, 50 ml of saltwater with 200 MBq of 99mTc and 13.6 of the reducing agent NaBH_4_ were mixed with a certain amount of dil-SFG. Then, the pH was set to 10. Water was added to bring the amount to 300 ml, and after 10 minutes of reaction at room temperature, samples were placed on chromatography paper (13 cm). The radiolabelling yield and in-vitro stability of the radioactive SF complex (99mTc-SF) were tested using paper chromatography and thin-layer chromatography [22]. The amount of free 99mTcO_4_- was measured with acetone, and the amount of reduced hydrolysed 99mTcO_2_ (colloid) was calculated with a mixture of ethanol, water, and ammonium hydroxide (2:5:1, v/v/v). The following formula was used to determine the labelling yield rate of the 99mTc-SF:

% labelling yield = 100 × [(% free 99mTcO_4_-) + (% reduced hydrolysed 99mTcO_2_ (colloid))]

**2.12. Preparation of transdermal 99mTc-SF gel**

The transdermal hydrogel for the 99mTc-SF (99mTc-SFG) was prepared with a 2% drug concentration using the same method as for formulating the SF gel.

***2.12.1. In-vivo biodistribution study of oral 99mTc-SF and transdermal 99mTc-SF gel***

The mice were separated randomly into two groups. Group A was given 33.3 mg of 5-FCY worth of 99mTc-SF by mouth through a gavage tube. The backs of the mice in Group B were covered with 99mTc-SFG containing 33.3 mg of Hal. Preliminary results showed that no radioactivity could be measured 8 hours after the oral formula had been given. At each time point, the animals in both groups were randomly separated into threes, weighed, and killed.

Jars already weighed and counted were filled with fresh blood samples. People used to think that blood made up 7% of the body’s weight. The brain, intestines, stomach, and liver were gathered, rinsed with normal saline, cleaned of any stuck-on tissue or fluid, and measured. The amount of radioactivity in each organ or tissue was measured with a shielded well-type gamma scintillation counter. As part of the amount applied or given, the percentage of radioactivity per gram (%R/g) was used to quantify the percentage of radiopharmaceutical taken up by each tissue or organ.

**2.13. Stability studies**

To see how SPLs would behave during storage, they were placed in a glass tube with a tight-fitting lid and refrigerated at 4 to 8°C for 1 month. The tubes were checked at 15 days and at the end of the storage time. On the first and last days of the stability study the PS, PDI, and ZP of the SPLs were noted.

**2.14. In-vitro antifungal activity**

The cup‒plate technique was employed to assess the antifungal efficacy of an optimized formulation. Sabouraud dextrose agar medium, consisting of 4 g of dextrose, 1.5 g of agar, and 1 g of mycological peptone, was utilized. To prepare the Sabouraud dextrose agar, 5.5 g of the medium was dissolved in 100 mL of distilled water under continuous heating and stirring. The solution was boiled for 1 minute to ensure complete dissolution of the powder, and the pH was maintained at 5.5. Subsequently, the prepared medium was autoclaved at 121°C for 15 minutes. The prepared agar medium was poured into three sterile glass petri dishes, each with a diameter of 93 mm, under aseptic conditions. The plates were left undisturbed for solidification. Once the agar medium had solidified, a sterile cork borer was used to create small holes on the surface of each agar plate. *C. albicans* was then inoculated by streaking it onto the agar surface. In each of the three petri dishes, small holes with a diameter of 8 mm were formed. In the first dish, 6 µg of 1% w/w SFG was placed in these holes; in the second dish, 6 µg of 5-FCY-SPL (0.25% w/w) was introduced into the holes; and in the third dish, 6 µg of 5-FCY was placed in the holes. Following the placement of these samples, the petri dishes were allowed to stand for a minimum of 30 minutes and were then incubated at a temperature of 25 ± 1°C for 24 hours. After 24 hours of incubation, the most consistent outer diameter of the inhibition zone was measured in millimeters using a vernier caliper (VWR International, Inc., Radnor, Pennsylvania, USA). This measurement included the diameter of the well. Each assay was conducted in triplicate.

**3. Results and Discussion**

**3.1. FT-IR analysis**

Figure 1 shows the FT-IR spectra of both the pure drug and the final mixture. The FT-IR spectrum of lactone C=O, the stretching vibration of the C–C double bond, and the peak of the enol-hydroxyl were 1783, 1683, and 1336 cm^-1^, respectively. The main absorption band of the characteristic peaks of pure 5-FCY did not disappear in the SPL mixture, which showed that the 5-FCY and the other ingredients did not interact.


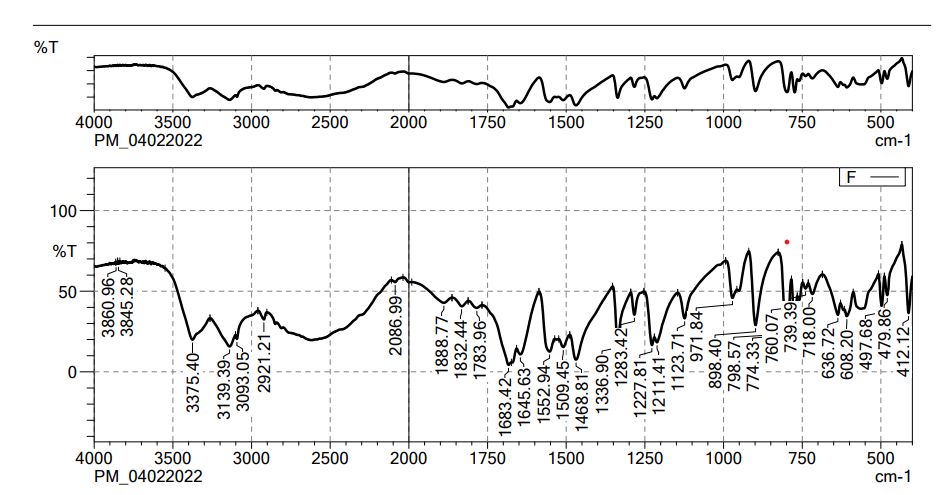


(a)


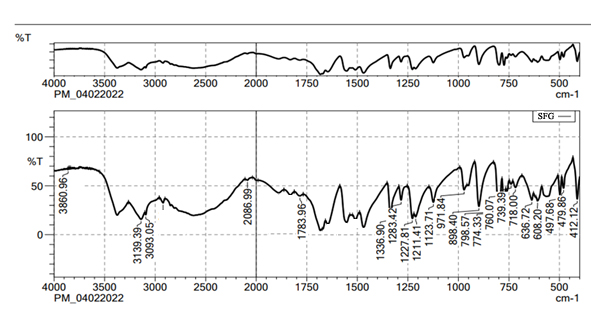


(b)

**Figure 1.** FT-IR spectra of (a) 5-FCY and (b) SPG formulation

**3.2. Optimization of preparation of 5-FCY-SPLs**

Design Expert 12 examined the quadratic response surfaces and how the different formulas were made by using a two-factor, five-level central composite design (Version 11.0, Stat-Ease Inc., Minneapolis, Minnesota, USA). This meant that 13 tests were done. Three-dimensional plots can show how two factors affect an answer and how these two factors affect each other. The usual plot of residuals shows that all of the models that were chosen were correct. In this case, the graph that could be seen was enough, so it was not necessary to use the mentioned statistical program. Statisticians can agree with the suggested model because all of the residuals for the chosen answers were more evenly distributed along a straight line. Table 2 summarizes the results for the 13 different formulas. All trial preparations had an entrapment efficiency of between 35% and 99% and a PS of between 25 and 620 nm. The results were examined, and statistical models such as ANOVA and fx were used to determine how the different factors affected each other. Based on the sum of squares (type I) and fit summary (adjusted and predicted R2 values) (see Table 2), the quadratic model was chosen for all of the results. The high-order polynomial quadratic model was chosen because the extra terms were easy to understand and the model does not overlap. For the PS and entrapment efficiency, the adjusted R2 values of 0.9593 and 0.9557 were nearly the same as the predicted R2 values of 0.8317 and 0.8429; the difference was less than 0.2. The signal-to-noise (S/N) ratio was measured with enough accuracy. Most of the time, a number more significant than 4 is better. The S/N ratios for the PS and EE were 26.5861 and 23.1059, respectively. This meant that there was more information than noise. Therefore, it was clear that the planning area was a good fit for the model. The plan was right because the model’s F-values for both answers were 29.85 and 43.35. Only 0.1% of the time could noise have caused the high F-value [16,25,26]. The coefficient of variation (CV) ensured that the plan could be used repeatedly. The accuracy of the most recent model was found to be 10% CV. The study found that the CV numbers were not too high, and this showed that the model was accurate and trustworthy. If the model does not fit well enough, it might not show all the data (Table 3). Therefore, there needs to be a lack of fit to show that the model’s equations are a good way to describe the results. The model picked for the study was good because neither of the results had a statistically significant *p*-value. Figure 2 shows how the factors that affected the results could be found by comparing the trial run with the residuals. Within the accepted range was a scattered trend, which showed a time-related variable in the background.

**Table 2.** Observed responses of projected extermintal runs for central composite design in optimizing the preparation of SPLs

|  |  | Factor 1 | Factor 2 | Response 1 | Response 2 |
| --- | --- | --- | --- | --- | --- |
| Std | Run | A: Span 60 | B: Tween 80 | PS | EE |
|  |  | mg | mg | nm | % |
| 7 | 9 | 200 | 71.7157 | 82 | 35 |
| 1 | 4 | 100 | 80 | 396 | 61 |
| 2 | 3 | 300 | 80 | 25 | 52 |
| 5 | 13 | 58.5786 | 100 | 788 | 78 |
| 13 | 1 | 200 | 100 | 253 | 45 |
| 10 | 2 | 200 | 100 | 253 | 48 |
| 11 | 5 | 200 | 100 | 254 | 49 |
| 9 | 6 | 200 | 100 | 259 | 51 |
| 12 | 7 | 200 | 100 | 261 | 47 |
| 6 | 11 | 341.421 | 100 | 450 | 85 |
| 3 | 12 | 100 | 120 | 620 | 58 |
| 4 | 8 | 300 | 120 | 480 | 99 |
| 8 | 10 | 200 | 128.284 | 385 | 76 |


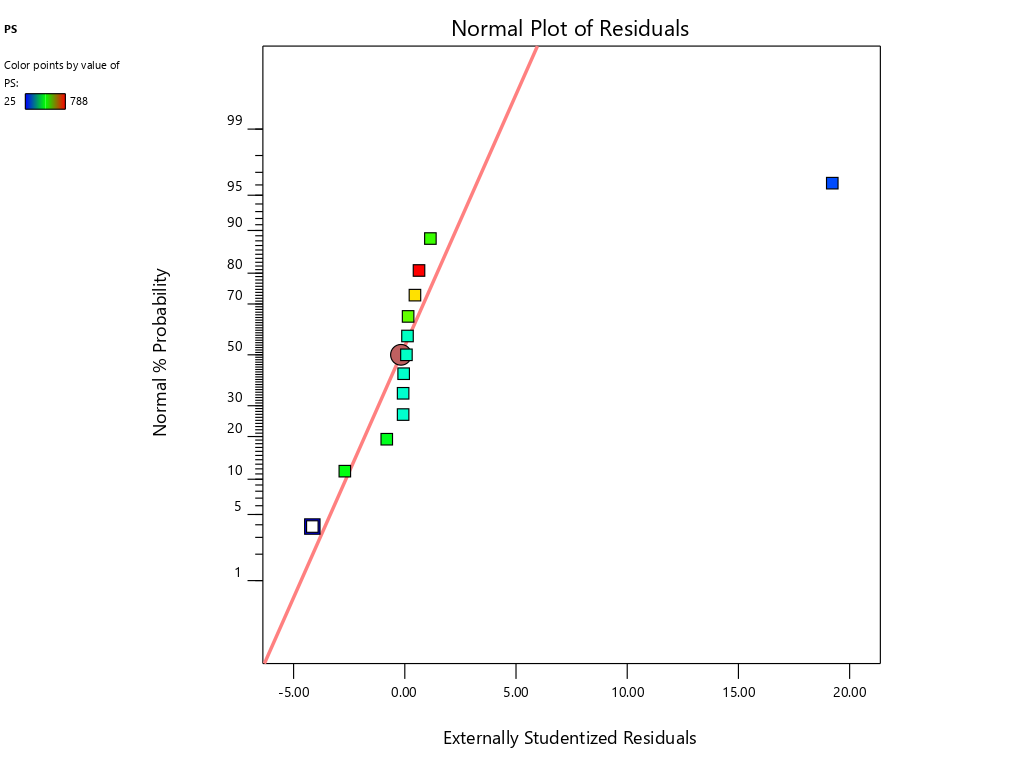

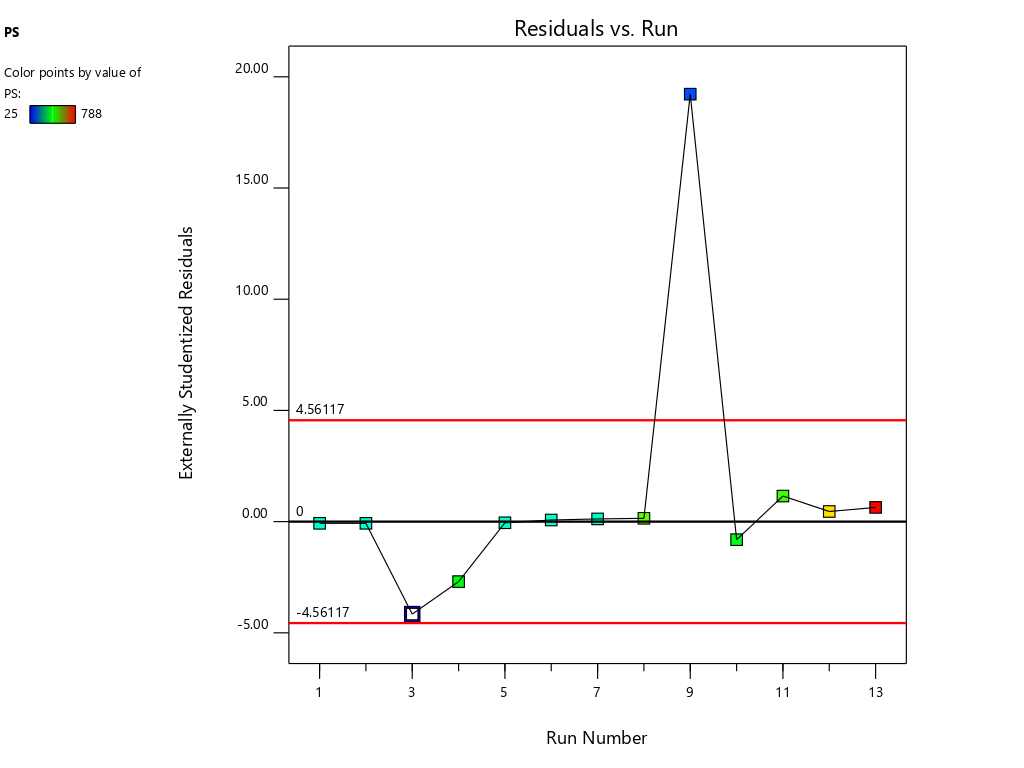
(a)


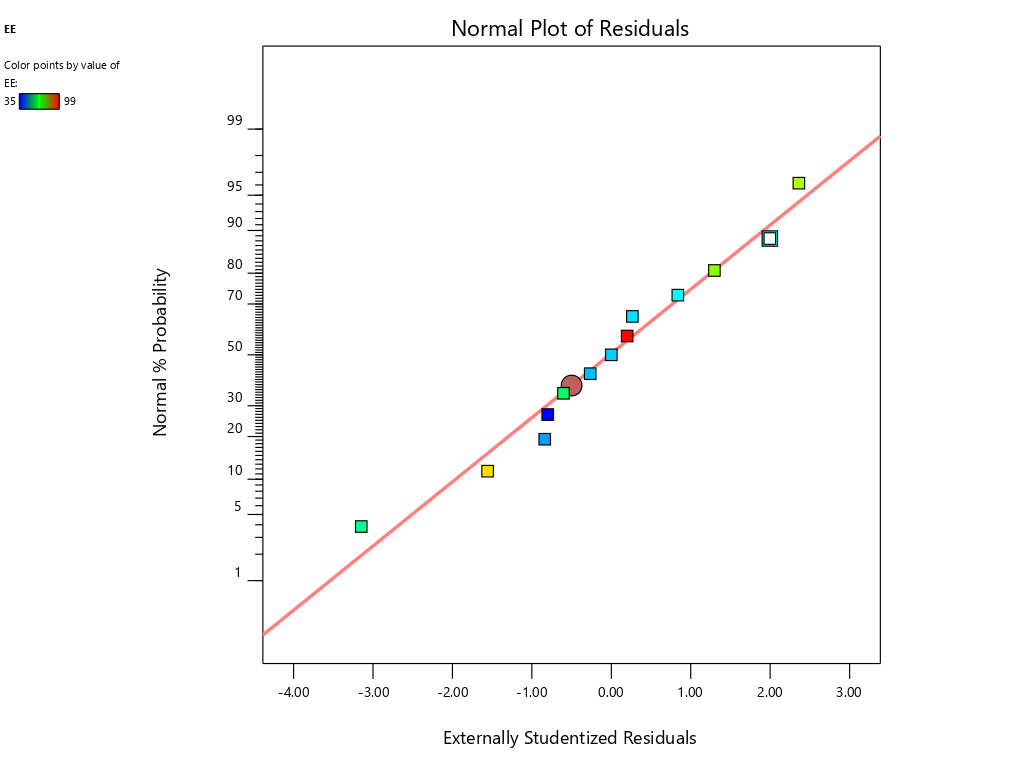

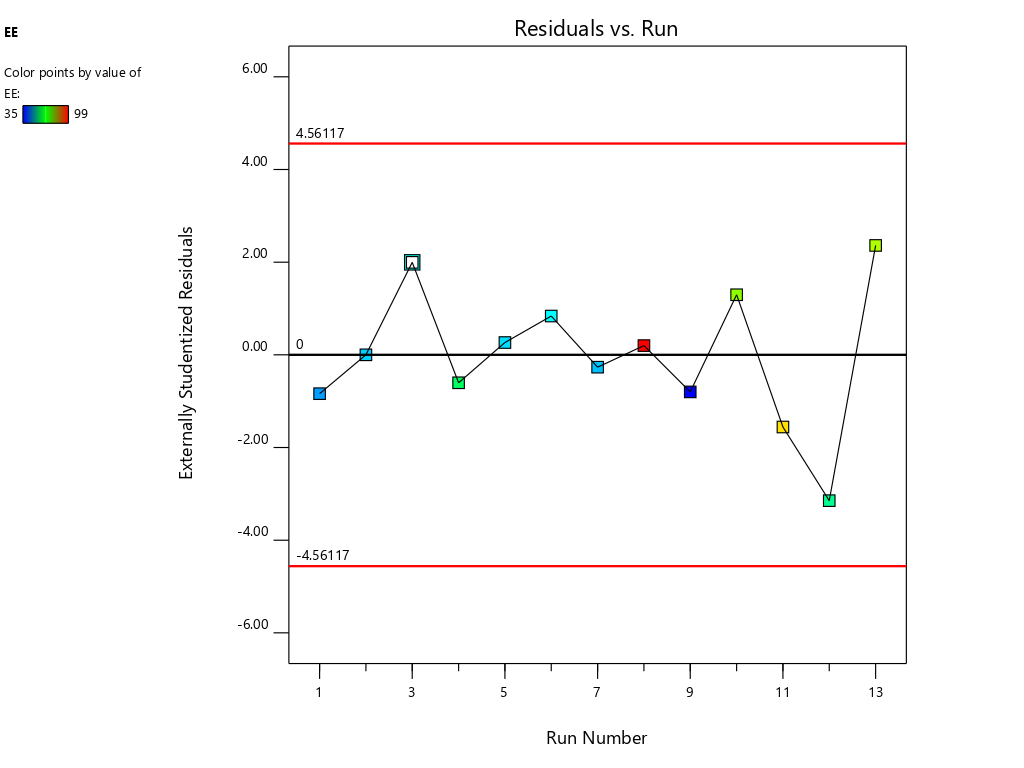
(b)

**Figure 2.** The normal plot for residuals and residuals vs. run for (a) PS and (b) EE

**Table 3.** Statistical summary of model

| Response | Source | R² | Adjusted R² | Predicted R² | Adequate Precision | Sequential *p*-value | Remarks |
| --- | --- | --- | --- | --- | --- | --- | --- |
| PS | Linear | 0.5335 | 0.4403 | 0.0527 | 26.5861 | .0221 |  |
|  | 2FI | 0.5594 | 0.4125 | 0.0540 |  | .4861 |  |
|  | Quadratic | **0.9762** | **0.9593** | **0.8317** |  | **< .0001** | Suggested |
|  | Cubic | 0.9917 | 0.9801 | 0.4755 |  | .0722 | Aliased |
| EE | Linear | 0.3654 | 0.2384 | ‒0.2076 | 23.1059 | .1029 |  |
|  | 2FI | 0.5157 | 0.3542 | ‒0.0788 |  | .1290 |  |
|  | Quadratic | **0.9742** | **0.9557** | **0.8429** |  | **< .0001** | Suggested |
|  | Cubic | 0.9947 | 0.9873 | 0.9617 |  | .0189 | Aliased |

**3.3. Study of the effect of independent variables or selected factors on PS and EE**

A small PS and its effect on drug delivery is one of the most important aspects of getting drugs to where they need to go. As the amount of Span 60 increases, the normal PS tends to go down. Tween 60 had a bigger effect on the PS, though. The PS got even better when Tween 80 played together more. Model terms are needed in ANOVA if the *p-value* is less than .0500 [27,28]. The model’s terms A, B, AB, and A2 were all important for the PS. The same pattern of essential factors can be seen in the EE. The experiment’s setup made it seem as though it could change the EE if all the important things worked together. Table 4 lists the ANOVA results. The 3D response graph in Figure 3a shows that high amounts of Span and least Tween can result in a smaller PS. High amounts of both factors are found in the area with the highest EE.

**Table 4.** ANOVA coefficients

|  | **Intercept** | **A** | **B** | **AB** | **A²** | **B²** |
| --- | --- | --- | --- | --- | --- | --- |
| **PS** | 256 | **‒123.626** | **138.438** | **57.75** | **170** | ‒22.75 |
| ***p*-values** |  | **< .0001** | **< .0001** | **.0281** | **< .0001** | .1949 |
| **EE** | 48 | **5.23744** | **12.7478** | **12.5** | **16.5** | 3.5 |
| ***p*-values** |  | **.0069** | **< .0001** | **.0004** | **< .0001** | .0507 |


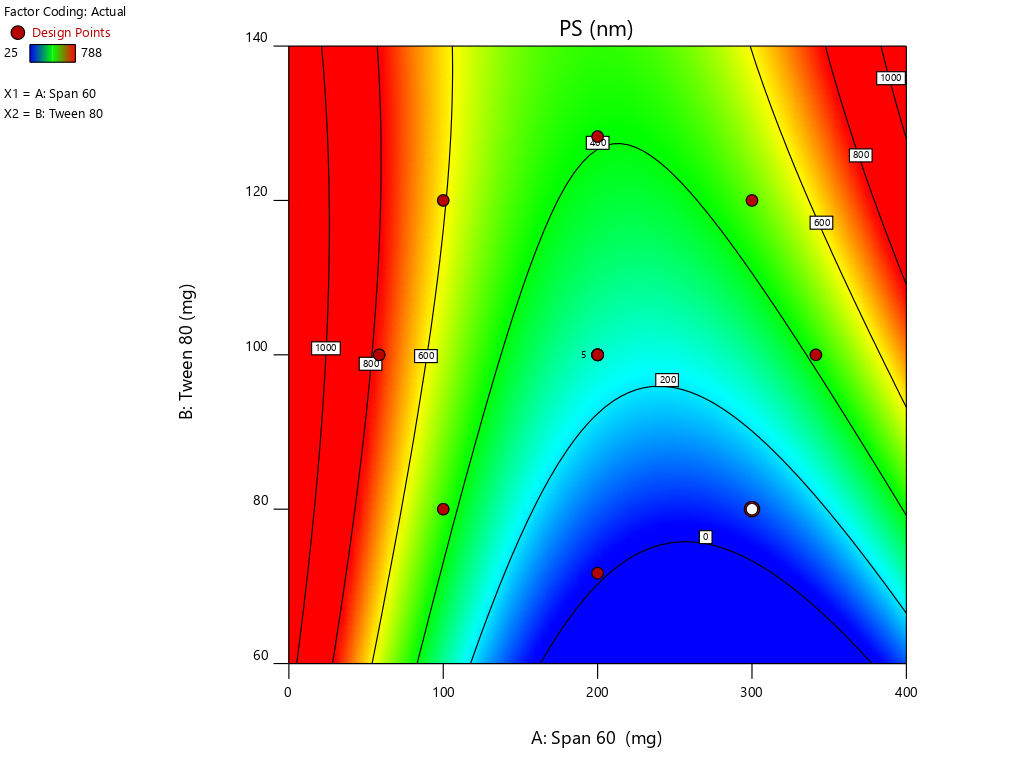

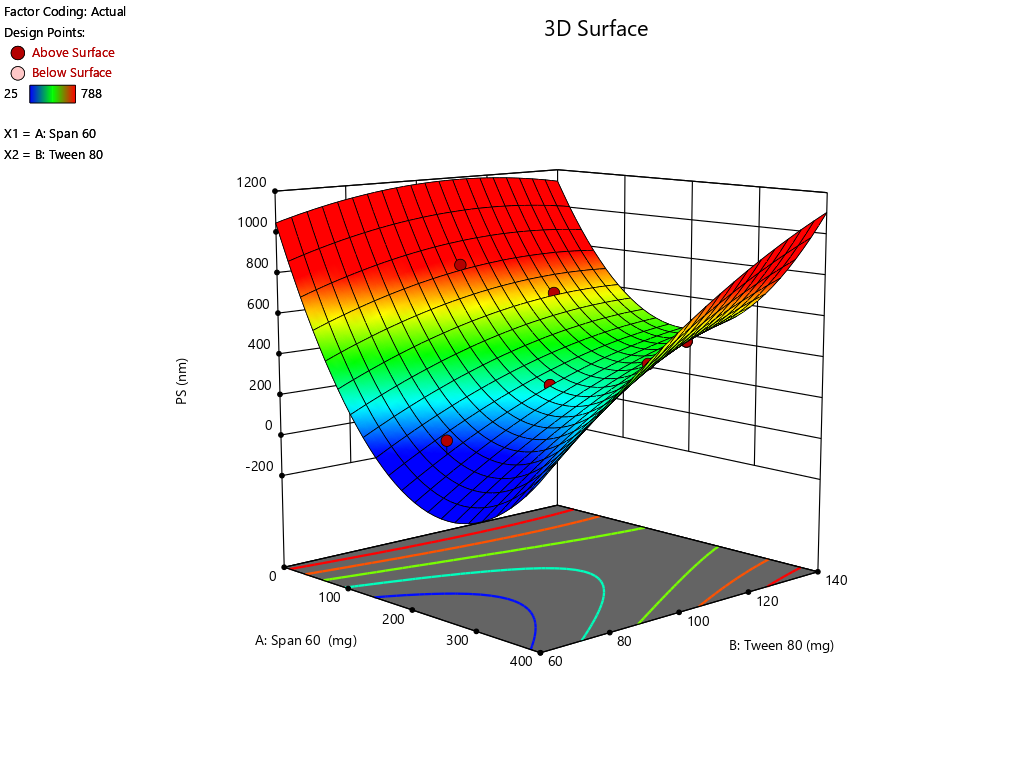
(a)
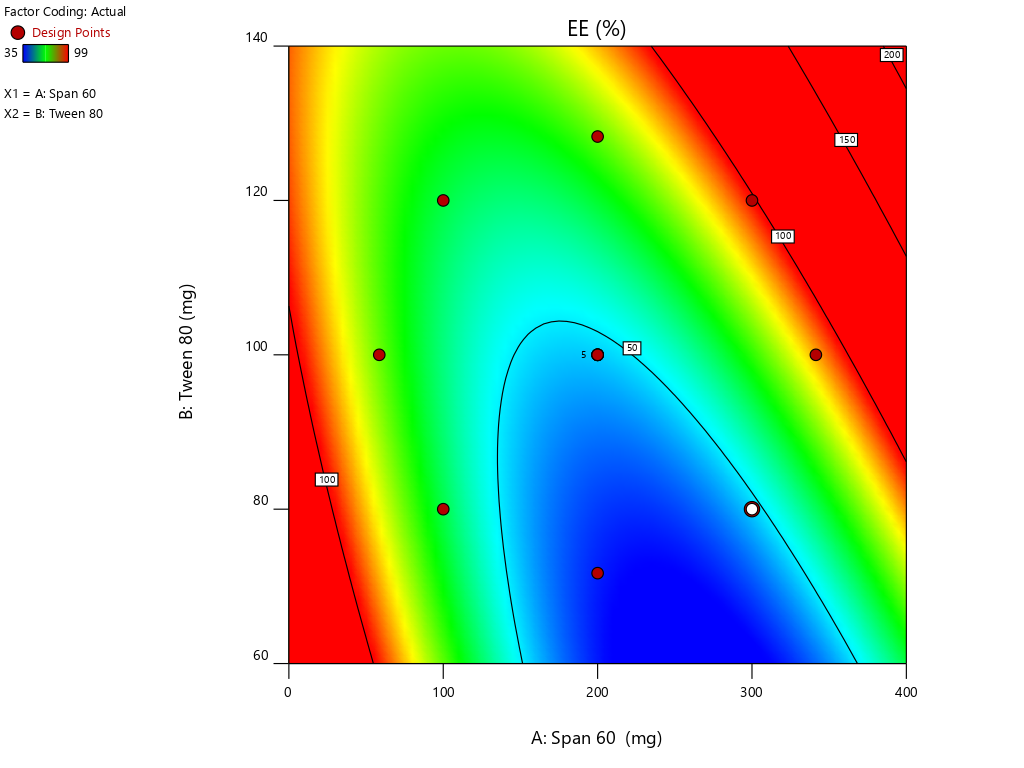

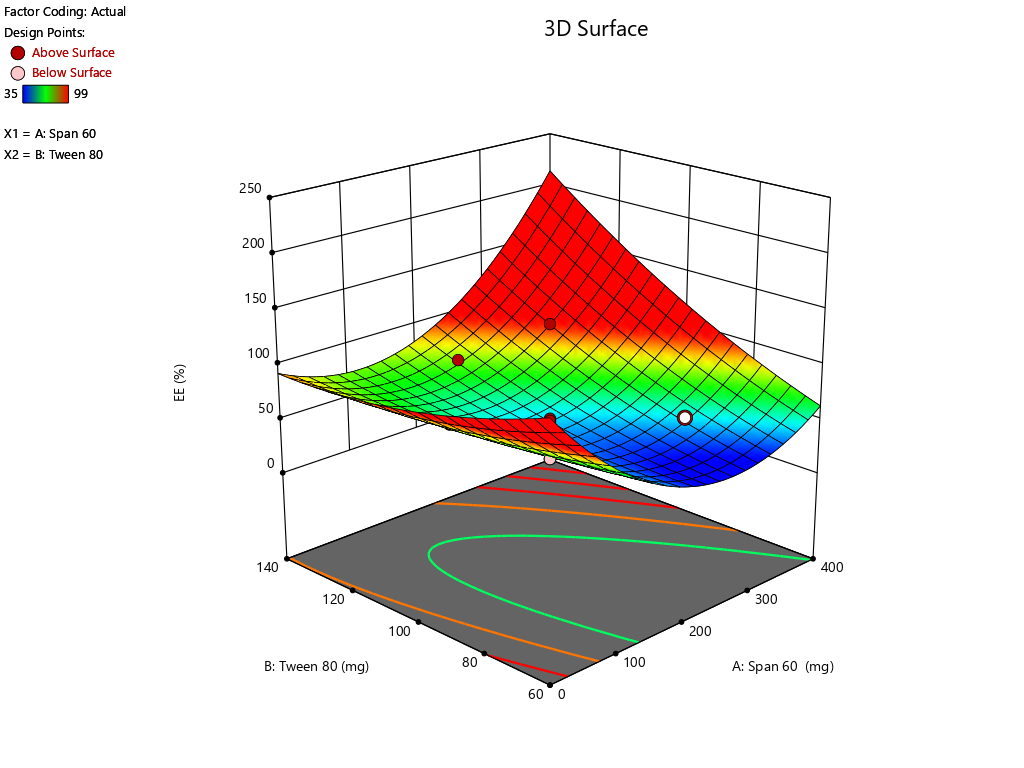
(b)

**Figure 3.** Countour and 3-D response surface plots for (a) PS and (b) EE

By employing a desirability function (D), various models generated from the experiment were optimized to their fullest potential. Specific boundaries were set to create an overlay graph, including the minimum PS and maximum ZP, among other criteria. The design space encompassed all the selected parameters. At the optimal ratios of the independent variables, the combined desirability plots for all the responses achieved the highest D-value of 0.638 (Figure 4a). Notably, the most critical responses coincided in the contour plot (see Figure 4b). Using this approach, a formulation meeting the conditions of an ideal formulation could be achieved by using 295.79 mg of Span 60 and 120.00 mg of Tween 80. Under these ideal ratios, the PS could be minimized to 464.57 nm and the EE could reach an impressive 96.379%. A new formula was made and tested using these predicted ideal ratios to see how well it worked. To affirm how the experiment was set up, the experiment’s results were compared with the numbers from the theory. It was found that the plan was correct less than 3% of the time. The PS was the same as what was found to be desirable, and the ZP was ‒34.5 mV, which shows that the composition was stable. The PDI is used to figure out how uniform a particle solution is on average, and higher PDI numbers mean that the particle sample has a wider range of sizes. The PDI can show how nanoparticles are clumped together, how the particles’ surfaces change, and how well they change as they move through the sample. When the PDI is 0.14, the method has been made as good as possible.


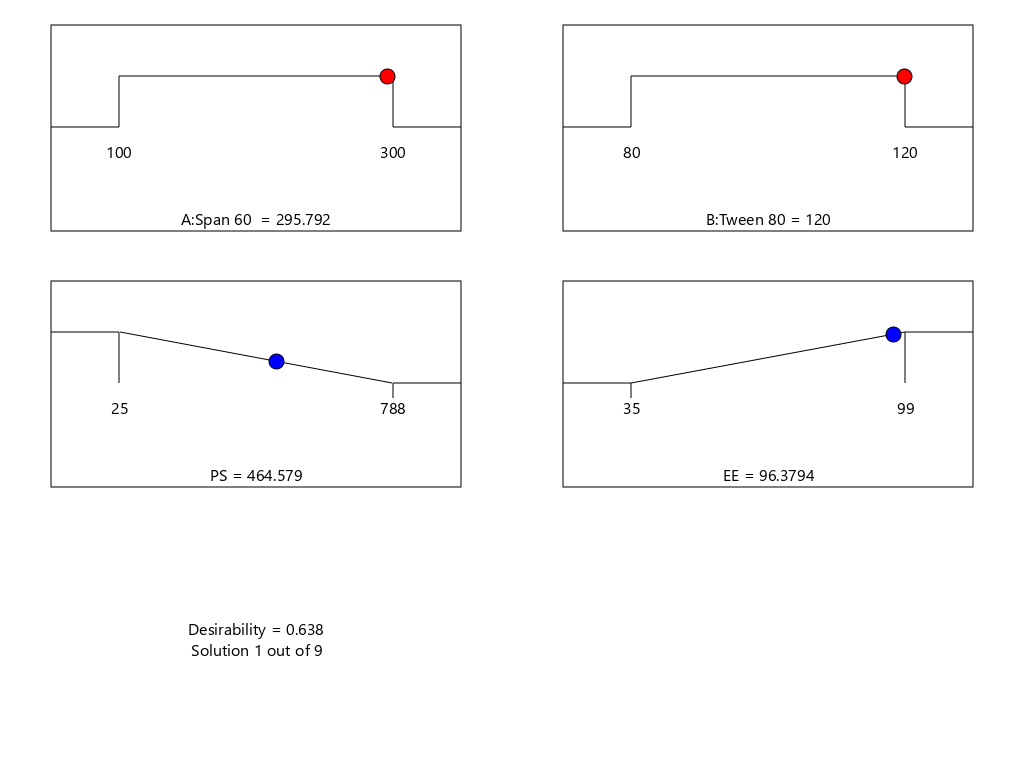
(a)


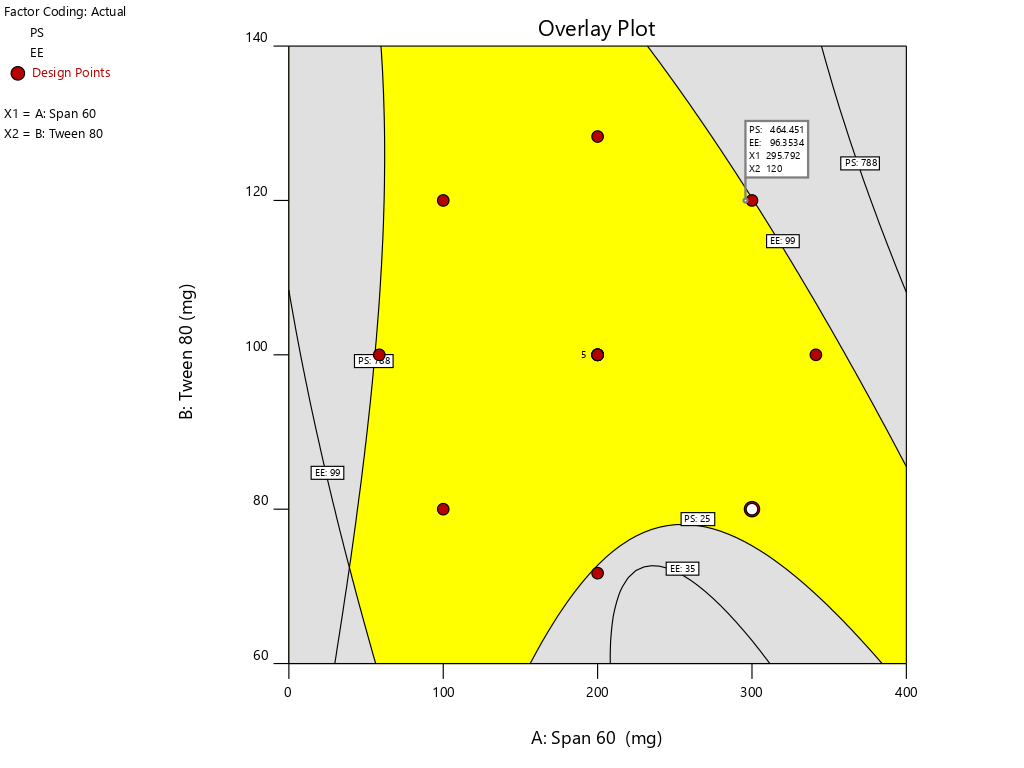
(b)

**Figure 4.** (a) Final optimization result and (b) overlay plot

**3.4. SPG characterization**

***3.4.1. Elasticity***

The deformability index (DI) and the extruded method were used to measure the elasticity of the SPG. The DI shows how well vesicles can fit through pores in the skin, which are smaller than the nanovesicles that have been made. The DI of the SPG was 10.58 ± 1.235, which means it was flexible and easy to shape. Hussain et al. (2017) found that when the edge activator Tween 80 was present in SPLs, the lipid bilayer became less stable, which made it more porous [29]. Tween is more flexible and stretchier, and therefore more able to pass through membranes, because it has non-bulky hydrocarbon chains and an unsaturated alkyl chain. Similar results were found by A. Manosroi et al. [30]. In their results, the DI values of the elastic niosomes were 13.76 and 3.44 times higher than the values of the conventional niosomes entrapped and not entrapped with the drug, respectively, indicating the higher flexibility of the elastic vesicle, especially, when it was entrapped with the drug [30].

**3.5. Ex-vivo permeation results**

Permeation data are presented in Figure 5. Notably, the drug was not detected for 4 hours when the SPG was measured and for 24 hours when the drug solution was measured; this implied a lag time. This delay could be attributed to the strong binding of the drug to the stratum corneum, as observed by Vaddi et al. (2002), which retards drug permeation. Furthermore, drug encapsulation in SPLs resulted in earlier detection of the drug in the permeation medium and an increased cumulative amount (Q) at various time intervals compared with the drug solution [31]. This phenomenon can be attributed to drug encapsulation within deformable vesicles, effectively shielding the drug from the stratum corneum. Additionally, the vesicles and the permeation enhancer used appeared to enhance drug permeation, resulting in a net increase in drug flux across the skin. At 12, 24, and 36 hours, both the cumulative amount (Q) and flux (J) were calculated for the 5-FCY solution and SPG (Table 5). It is clear that all of the SPL formulations consistently exhibited significantly higher Q-values and J-values than the drug solution at all time intervals, indicating a substantial improvement in 5-FCY flux across the skin. Notably, the SPG formulation displayed the highest values in Q12, Q24, Q36, J12, and J24. Given the importance of ex-vivo permeation data for selection criteria, SPG was identified as the optimal formulation for further investigation in in-vivo studies.

**Figure 5.** Permeation plots for 5-FCY from SPG

**Table 5.** Ex-vivo permeation parameters

|  | Q (lg/cm^2^)c | | | J (mg/cm^2^/h)c | | |
| --- | --- | --- | --- | --- | --- | --- |
|  | Q_12_ | Q_24_ | Q_36_ | J_12_ | J_24_ | J_36_ |
| Drug solution | 21.52±7.12 | 39.01±7.83 | 41.41±8.45 | 0.2±0.03 | 0.3±0.04 | 0.4±0.02 |
| SPG | 172.45±45.96 | 186.34±68.30 | 215.87±45.21 | 2.8±0.4 | 3.0±0.2 | 3.9±0.2 |

**3.6. In-vitro release of 5-FCY from SPG**

Figure 6 illustrates the release profiles of 5-FCY from SPG and the drug solution over time. Incorporating SPLs into the hydrogel matrix resulted in a more prolonged release profile than that of the drug solution, which released all of its drug in just under 2 hours. The release curves of FVS in various SNVs exhibited a clear biphasic pattern. This biphasic behavior consisted of a rapid release of drug adsorbed on the surface during the initial 2 hours, termed the “initial phase,” followed by a slower sustained release. Previous ex-vivo studies have indicated that the rapid release observed in Span-based SPLs may be attributed to their unsaturated alkyl chains, which can lead to drug leakage. Additionally, the lower phase transition temperature of Spans could contribute to the increased release rate observed. Since the release study was conducted at 32 ± 0.5°C, it is plausible that the higher transition temperature of the Tween-based vesicles, making them more gel-like, was responsible for their comparatively lower release rates. The release data for 5-FCY from SPG was best described by the Higuchi diffusion model, as determined by a kinetic study, which yielded the highest R2 value. Several studies, including those by Mahmoud et al. (2017) and Aboud et al. (2018), employing Higuchi’s square root model, reported controlled drug release from vesicular systems. We employed the Korsmeyer‒Peppas model to gain a deeper understanding of the drug release mechanisms. This model aided in elucidating how various drugs are released. In the Korsmeyer–Peppas equation, the n-values correspond to Fickian (diffusional) and zero-order release kinetics when they are 0.5 and 1, respectively, while for non-Fickian (abnormal) release, the value is 0.5 < n < 1. Our study found that the n-values for the different dispersions were all approximately 0.73, indicative of non-Fickian drug diffusion and an abnormal drug release pattern, where the drug diffusion may be intertwined with the expansion of lipid bilayers.

**Figure 6.** Drug release profile of pure drug solution and SFG formulation.

**3.7. Effect of storage conditions on physical and chemical properties of SPLs**

After 1 month of the stability study, no discernible change in color of the SPLs was observed. Statistical analysis involved the utilization of a one-way ANOVA and a post-hoc test in the GraphPad program to compare the mean and standard deviation. It was determined that, except for aggregation, the formulation did not significantly alter the PS, PDI, or ZP (*p* > .05) (Table 6). Furthermore, the EE exhibited a minimal variation (*p* > .05) (96.37% to 94.89%), indicating the stability of the 5-FCY in the formulation.

Table 6. Stability parameters of SPLs

| **Parameter** | **Initial** | **Refrigeration Condition** | |
| --- | --- | --- | --- |
|  |  | **15 days** | **30 days** |
| Physical appearance | Complies | Complies | Complies |
| PS (nm) | 464.57 | 463.21 | 462.86 |
| ZP (mV) | 34.5 | 34.1 | 33.9 |
| PDI | 0.14 | 0.15 | 0.15 |

**3.8. In-vitro antifungal activity**

The agar diffusion method was used for this microbiological study. Results showed that the SPG combination in the SPL significantly inhibited the growth of C. *albicans* as compared with the SPL and that the antifungal activity was enhanced through a conversion into a gel form. The mean diameters of the zones of inhibition against *C.* *albicans* were SFG 19.44 ± 0.89 mm, 5-FCY-SPL 9.40 ± 0.68, and 5-FCY 4.21 ± 0.78 mm. The zones of inhibition for SPG were higher, and this was due to a synergistic effect of the drug combined with the SPL as shown in Figure 7.


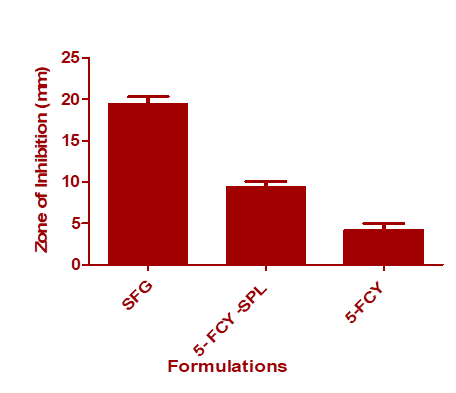


**Figure 7.** In-vitro antifungal activity of 5-FCY, 5-FCY-SPL, and SFG

**3.9. In-vivo distribution study**

Figure 8 illustrates the radioactive complex’s liver uptake and blood levels following the oral administration of 99mTc-5-FCY and the transdermal application of the 99m SFG at various time intervals. Initially, and up to the 3-hour mark, the liver’s uptake of the radioactive complex was significantly higher after oral administration. This observation underscored the extensive first-pass metabolism associated with oral dosing. It explained why the radioactive complex remained undetectable in various organs 24 hours after oral administration, even though it was detected 48 hours after transdermal application. However, 6 hours after transdermal application, the liver’s uptake of the radioactive complex surpassed that associated with oral administration, and it peaked at 12 hours. The elevated levels of the radioactive complex detected in the liver after transdermal application may be attributed to its localization, which depends on both the formulation and the application site. When comparing the calculated area under the curve from time zero to 8 hours (AUC0–24) following transdermal application and oral administration, it is evident that the liver levels were significantly lower after transdermal application. This lower systemic and hepatic exposure is favorable for controlling extrapyramidal side effects and the hepatic first-pass metabolism associated with oral administration.

Additionally, transdermal application resulted in a significantly longer time to reach maximum concentration (Tmax) than oral administration. Deformable vesicles such as porcine vascular endothelial cells (PECs) are known for their prolonged circulation times following transdermal application, and this helps protect 5-FCY in the bloodstream before its release. Regarding blood levels, transdermal application resulted in marked fluctuations. It exhibited slower Tmax values of 6 hours and 12 hours. After 6 hours, a decline in concentration was observed, followed by an increase at 12 hours and a negative slope. This phenomenon can be explained by the fact that the skin at the administration site acts as a reservoir for the drug and the SFG, leading to a pulsatile release pattern. This pattern is beneficial for maintaining high drug levels for extended periods, although it was not evident in the in-vitro release profiles. The variation may be attributed to the differing rates and relative amounts by which the radioactive complex reaches the systemic and lymphatic circulations after transdermal application. Despite lower brain levels of the drug following transdermal application for the initial 3 hours, the drug concentration reached its maximum level within the subsequent 3 hours. This suggests evidence of sustained drug delivery from the transdermal formulation, an effect that may enable reductions in dosage size and frequency, which could mitigate side effects and enhance patient compliance.

**Figure 8.** Biodistribution studies of radiolabelled formulations by oral and topical delivery

**4. Conclusions**

A statistically optimized way of injecting ethanol was used to make 5-FCY‒loaded SPLs that worked well. When the formulas were adjusted, the PS was 464.57 nm and the EE was 96.379%. The difference between what was predicted and what actually was found was less than 2%, which shows that the chosen plan was good. The improved formula also showed that the ZP and PDI were, respectively, ‒34.5 mV and 0.14. This indicates that the recipe does not change. The SFG was a flexible substance, let the drug through, and had a good drug release curve and in-vitro antifungal activity. The biodistribution study showed that the drug was released slowly and in more bursts from the transdermal hydrogel. Lastly, an SPL-loaded topical hydrogel can guarantee therapeutic gains by making a drug more bioavailable.

**Author Contributions:**

Conceptualization, N.R.N., A.Y.A, A.A.; methodology, M.K., A.S.H, K.Z., K.H.; software, A.Y.S., N.R.N., M.K., W.A.A.; validation, M.K, K.H., A.A.A., M.A.; formal analysis and investigation, N.R.N., M.K., R.K.; resources, W.A.A., R.K., A.S.H.; data curation, M.K., A.A.A.; M.A.; writing—original draft preparation, N.R.N., A.A.; writing—review and editing, M.A., M.K., A.Y.S.; visualization and supervision, N.R.N., A.A.; project administration, W.A., M.A., A.Y.S.; funding acquisition, W.A., R.K.,A.S.H. All authors have read and agreed to the published version of the manuscript.

**Funding:** The Deanship of Scientific Research (DSR) at King Abdulaziz University, Jeddah, Saudi Arabia, funded this project under grant no. RG-4-166-43.

**Institutional Review Board Statement:** The animal study protocol was approved by the Animal Ethics Committee of Cairo Agriculture for Experimental Animals, Cairo, Egypt, Approval No. 05-07-23.

**Informed Consent Statement:** Not applicable.

**Data Availability Statement:** The data used to support the findings of this study are included in the article.

**Acknowledgments:** The Deanship of Scientific Research (DSR) at King Abdulaziz University (KAU), Jeddah, Saudi Arabia, has funded this project under grant no. RG-4-166-43.

**Conflicts of Interest:** The authors declare no conflicts of interest.

**References**

1. Hidron, A.; Edwards, J.; Patel, J.; Horan, T. update: antimicrobial-resistant pathogens associated with healthcare-associated infections: annual summary of data reported to the National Healthcare. Infect Control Hosp. 2008, 29.

2. Morgan, J.; Meltzer, M.I.; Plikaytis, B.D.; Sofair, A.N.; Huie-White, S.; Wilcox, S.; Harrison, L.H.; Seaberg, E.C.; Hajjeh, R.A.; Teutsch, S.M. Excess Mortality, Hospital Stay, and Cost Due to Candidemia: A Case-Control Study Using Data From Population-Based Candidemia Surveillance. Infect. Control Hosp. Epidemiol. 2005, 26, doi:10.1086/502581.

3. Wisplinghoff, H.; Bischoff, T.; Tallent, S.M.; Seifert, H.; Wenzel, R.P.; Edmond, M.B. Nosocomial bloodstream infections in US hospitals: Analysis of 24,179 cases from a prospective nationwide surveillance study. Clin. Infect. Dis. 2004, 39, doi:10.1086/421946.

4. Cowen, L.E.; Steinbach, W.J. Stress, drugs, and evolution: The role of cellular signaling in fungal drug resistance. Eukaryot. Cell 2008, 7.

5. Bu, Q.R.; Bao, M.Y.; Yang, Y.; Wang, T.M.; Wang, C.Z. Targeting Virulence Factors of Candida albicans with Natural Products. Foods 2022, 11.

6. Kumamoto, C.A.; Vinces, M.D. Contributions of hyphae and hypha-co-regulated genes to Candida albicans virulence. Cell. Microbiol. 2005, 7.

7. Lo, H.J.; Köhler, J.R.; Didomenico, B.; Loebenberg, D.; Cacciapuoti, A.; Fink, G.R. Nonfilamentous C. albicans mutants are avirulent. Cell 1997, 90, doi:10.1016/S0092-8674(00)80358-X.

8. Naglik, J.R.; Challacombe, S.J.; Hube, B. Candida albicans Secreted Aspartyl Proteinases in Virulence and Pathogenesis . Microbiol. Mol. Biol. Rev. 2003, 67, doi:10.1128/mmbr.67.3.400-428.2003.

9. Patra, J.K.; Das, G.; Fraceto, L.F.; Campos, E.V.R.; Rodriguez-Torres, M.D.P.; Acosta-Torres, L.S.; Diaz-Torres, L.A.; Grillo, R.; Swamy, M.K.; Sharma, S.; et al. Nano based drug delivery systems: Recent developments and future prospects. J. Nanobiotechnology 2018, 16.

10. Farghaly, D.A.; Aboelwafa, A.A.; Hamza, M.Y.; Mohamed, M.I. Topical Delivery of Fenoprofen Calcium via Elastic Nano-vesicular Spanlastics: Optimization Using Experimental Design and In Vivo Evaluation. AAPS PharmSciTech 2017, 18, doi:10.1208/s12249-017-0771-8.

11. Islam, M.M.; Ramesh, V.H.; Bhavani, D.; Goudanavar, P.S.; Naveen, N.R.; Ramesh, B.; Fattepur, S.; Shiroorkar, N.; Habeebuddin, M.; Meravanige, G.; et al. Optimization of process parameters for fabrication of electrospun nanofibers containing neomycin sulfate and Malva sylvestris extract for a better diabetic wound healing. https://doi.org/10.1080/10717544.2022.2144963 2022, 29, 3370–3383, doi:10.1080/10717544.2022.2144963.

12. Sreeharsha, N.; Prasanthi, S.; Mahalakshmi, S.V.V.N.S.; Goudanavar, P.S.; Naveen, N.R.; Gowthami, B.; Fattepur, S.; Meravanige, G.; Asdaq, S.M.B.; Anwer, M.K.; et al. Enhancement of Anti-Tumoral Properties of Paclitaxel Nano-Crystals by Conjugation of Folic Acid to Pluronic F127: Formulation Optimization, In Vitro and In Vivo Study. Mol. 2022, Vol. 27, Page 7914 2022, 27, 7914, doi:10.3390/MOLECULES27227914.

13. Grangeia, H.B.; Silva, C.; Simões, S.P.; Reis, M.S. Quality by design in pharmaceutical manufacturing: A systematic review of current status, challenges and future perspectives. Eur. J. Pharm. Biopharm. 2020, 147.

14. Rizg, W.Y.; Naveen, N.R.; Kurakula, M.; Bukhary, H.A.; Safhi, A.Y.; Alfayez, E.; Sindi, A.M.; Ali, S.; Murshid, S.S.; Hosny, K.M. QbD Supported Optimization of the Alginate-Chitosan Nanoparticles of Simvastatin in Enhancing the Anti-Proliferative Activity against Tongue Carcinoma. Gels 2022, 8, 103.

15. Sreeharsha, N.; Rajpoot, K.; Tekade, M.; Kalyane, D.; Nair, A.B.; Venugopala, K.N.; Tekade, R.K. Development of Metronidazole Loaded Chitosan Nanoparticles Using QbD Approach—A Novel and Potential Antibacterial Formulation. Pharm. 2020, Vol. 12, Page 920 2020, 12, 920, doi:10.3390/PHARMACEUTICS12100920.

16. Rizg, W.Y.; Naveen, N.R.; Kurakula, M.; Bukhary, H.A.; Safhi, A.Y.; Alfayez, E.; Sindi, A.M.; Ali, S.; Murshid, S.S.; Hosny, K.M. QbD Supported Optimization of the Alginate-Chitosan Nanoparticles of Simvastatin in Enhancing the Anti-Proliferative Activity against Tongue Carcinoma. Gels (Basel, Switzerland) 2022, 8, doi:10.3390/GELS8020103.

17. Mokale, V.J.; Patil, H.I.; Patil, A.P.; Shirude, P.R.; Naik, J.B. Formulation and optimisation of famotidine proniosomes: an in vitro and ex vivo study. J. Exp. Nanosci. 2016, 11, doi:10.1080/17458080.2015.1030711.

18. Sallam, N.M.; Sanad, R.A.B.; Ahmed, M.M.; Khafagy, E.S.; Ghorab, M.; Gad, S. Impact of the mucoadhesive lyophilized wafer loaded with novel carvedilol nano-spanlastics on biochemical markers in the heart of spontaneously hypertensive rat models. Drug Deliv. Transl. Res. 2021, 11, 1009–1036, doi:10.1007/S13346-020-00814-4/METRICS.

19. Mazyed, E.A.; Helal, D.A.; Elkhoudary, M.M.; Abd Elhameed, A.G.; Yasser, M. Formulation and optimization of nanospanlastics for improving the bioavailability of green tea epigallocatechin gallate. Pharmaceuticals 2021, 14, 1–30, doi:10.3390/ph14010068.

20. Kurakula, M.; Naveen N., R.; Patel, B.; Manne, R.; Patel, D.B. Preparation, Optimization and Evaluation of Chitosan-Based Avanafil Nanocomplex Utilizing Antioxidants for Enhanced Neuroprotective Effect on PC12 Cells. Gels 2021, 7, doi:10.3390/gels7030096.

21. Mallappa, D.P.; Chelsea, F.R.; Ratnakar, R.P.; Panchakshari, G.A.; Shivamurthi, M.V.; Uppinangady, B.S. Development and Characterization of Mucoadhesive Buccal Gel Containing Lipid Nanoparticles of Triamcinolone Acetonide. Indian J. Pharm. Educ. Res. 2020, 54, s505–s511, doi:10.5530/ijper.54.3s.149.

22. Fahmy, A.M.; El-Setouhy, D.A.; Ibrahim, A.B.; Habib, B.A.; Tayel, S.A.; Bayoumi, N.A. Penetration enhancer-containing spanlastics (PECSs) for transdermal delivery of haloperidol: In vitro characterization, ex vivo permeation and in vivo biodistribution studies. Drug Deliv. 2018, 25, 12–22, doi:10.1080/10717544.2017.1410262.

23. Gad, H.A.; El-Nabarawi, M.A.; Abd El-Hady, S.S. Formulation and evaluation of PLA and PLGA in situ implants containing secnidazole and/or doxycycline for treatment of periodontitis. AAPS PharmSciTech 2008, 9, 878–884, doi:10.1208/S12249-008-9126-9.

24. Fang, J.Y.; Yu, S.Y.; Wu, P.C.; Huang, Y. Bin; Tsai, Y.H. In vitro skin permeation of estradiol from various proniosome formulations. Int. J. Pharm. 2001, 215, doi:10.1016/S0378-5173(00)00669-4.

25. Sreeharsha, N.; Naveen, N.R.; Anitha, P.; Goudanavar, P.S.; Ramkanth, S.; Fattepur, S.; Telsang, M.; Habeebuddin, M.; Answer, M.K. Development of Nanocrystal Compressed Minitablets for Chronotherapeutic Drug Delivery. Pharm. 2022, Vol. 15, Page 311 2022, 15, 311, doi:10.3390/PH15030311.

26. Aldawsari, H.M.; Naveen, N.R.; Alhakamy, N.A.; Goudanavar, P.S.; Rao, G.K.; Budha, R.R.; Nair, A.B.; Badr-Eldin, S.M. Compression-coated pulsatile chronomodulated therapeutic system: QbD assisted optimization. Drug Deliv. 2022, 29, 2258–2268, doi:10.1080/10717544.2022.2094500.

27. Bakhaidar, R.B.; Naveen, N.R.; Basim, P.; Murshid, S.S.; Kurakula, M.; Alamoudi, A.J.; Bukhary, D.M.; Jali, A.M.; Majrashi, M.A.; Alshehri, S.; et al. Response Surface Methodology (RSM) Powered Formulation Development, Optimization and Evaluation of Thiolated Based Mucoadhesive Nanocrystals for Local Delivery of Simvastatin. Polymers (Basel). 2022, 14, 1–17, doi:10.3390/polym14235184.

28. Alhakamy, N.A.; Naveen, N.R.; Gorityala, S.; Kurakula, M.; Hosny, K.M.; Safhi, A.Y.; Bukhary, D.M.; Bukhary, H.A.; Sabei, F.Y.; Mushtaq, R.Y.; et al. Development of Novel S-Protective Thiolated-Based Mucoadhesive Tablets for Repaglinide: Pharmacokinetic Study. Polym. 2022, Vol. 14, Page 3529 2022, 14, 3529, doi:10.3390/POLYM14173529.

29. Hussain, A.; Singh, S.; Sharma, D.; Webster, T.J.; Shafaat, K.; Faruk, A. Elastic liposomes as novel carriers: recent advances in drug delivery. Int. J. Nanomedicine 2017, 12, 5087–5108, doi:10.2147/IJN.S138267.

30. Manosroi A, Jantrawut P, Manosroi J. Anti-inflammatory activity of gel containing novel elastic niosomes entrapped with diclofenac diethylammonium. Int J Pharm. 2008 6;360(1-2):156-63. doi: 10.1016/j.ijpharm.2008.04.033. Epub 2008 Apr 26. PMID: 18539416..

31. Vaddi, H.K.; Ho, P.C.; Chan, S.Y. Terpenes in propylene glycol as skin-penetration enhancers: permeation and partition of haloperidol, Fourier transform infrared spectroscopy, and differential scanning calorimetry. J. Pharm. Sci. 2002, 91, 1639–1651, doi:10.1002/JPS.10160.
